# A Cross-Sectional Comparison of Patient Information Guides Generated by ChatGPT Versus Google Gemini for Alzheimer’s Disease, Parkinsonism, and Migraine

**DOI:** 10.7759/cureus.84507

**Published:** 2025-05-20

**Authors:** Aayush Chaulagain, Savvy Aujla, Archana Priyadarsini, Aarav K Godavarthi, Usman Yaqoob

**Affiliations:** 1 Internal Medicine, Shaheed Ziaur Rahman Medical College and Hospital, Bogra, BGD; 2 Orthopedics, Government Medical College, Amritsar, Amritsar, IND; 3 Internal Medicine, Dr. Somervell Memorial CSI Medical College & Hospital, Thiruvananthapuram, IND; 4 Internal Medicine, Shadow Creek High School, Pearland, USA; 5 Internal Medicine, Rowan-Virtua School of Osteopathic Medicine, Stratford, USA

**Keywords:** alzheimer’s disease, artificial intelligence, chatgpt, educational tool, google gemini, migraine, parkinsonism, patient education brochure

## Abstract

Introduction

This study aims to compare the characteristics of educational brochures produced by two large language models for common neurological diseases such as migraine (MIG), Parkinson’s disease, and Alzheimer’s disease (AD). Despite the enthusiasm surrounding these technologies, there remains a critical need to systematically investigate their effectiveness, usability, and impact within healthcare contexts. This cross-sectional study investigates patient education brochures for AD, Parkinsonism, and MIG, emphasizing the emerging role of AI-driven tools, such as ChatGPT and Google Gemini.

Methods

Utilizing a patient information brochure approach, we compared responses generated by ChatGPT and Google Gemini, which, at the time of the study, were the two well-known and well-developed AI tools, by using the prompt “This cross-sectional study investigates patient education brochures for Alzheimer’s disease, Parkinsonism, and migraine, emphasizing the emerging role of AI-driven tools, such as ChatGPT and Google Gemini.” Readability and reliability were assessed using the Flesch-Kincaid calculator and Modified DISCERN Score, respectively. Statistical analysis was conducted using R software version 4.3.2.

Results

The results show no significant differences in mean word and sentence counts between the models, although Google Gemini produced shorter texts with fewer sentences (p = 0.04). Both models had similar average words per sentence (p = 0.97) and syllables per word (p = 0.28), but Google Gemini’s texts were slightly more complex (ease score p = 0.29). Google Gemini’s outputs were also more original, with lower similarity scores (p = 0.04). Pearson correlation coefficients indicated a moderate negative, though statistically insignificant, relationship between ease and reliability scores for both models.

Conclusions

While Google Gemini produced shorter and potentially more original content, no significant superiority of one AI tool over the other was observed, suggesting the need for ongoing refinement to optimize patient education materials for neurological conditions.

## Introduction

Alzheimer’s disease (AD), Parkinsonism, and migraine (MIG) represent distinct yet intricate neurological conditions. AD is a progressive neurodegenerative disorder characterized by cognitive decline, memory impairment, and behavioral changes, primarily driven by the accumulation of beta amyloid peptides forming neuritic plaques and hyperphosphorylation of Tau protein leading to neurofibrillary tangles in key brain regions [1]. Parkinsonism refers to a complex clinical syndrome characterized by a range of symptoms, including rigidity, bradykinesia, tremor, and postural instability [2]. MIG, on the other hand, is a multifaceted disorder influenced by genetics, marked by recurring episodes of moderate-to-severe headaches, typically affecting one side of the head and often accompanied by symptoms like nausea, as well as heightened sensitivity to light and sound [3].

Patient education is a fundamental pillar in healthcare, allowing individuals to detect potential complications early on and seek timely medical intervention. Furthermore, it fosters medication adherence, a critical aspect in managing chronic illnesses and mitigating adverse health outcomes, thereby contributing significantly to improved overall health and decreased mortality rates.

AI tools are incredibly valuable for patient education because they provide accurate and constantly updated information about a wide range of diseases. They ensure that patients can access the right information anytime and anywhere, enabling them to make informed decisions about their health. ChatGPT, an OpenAI creation, is an advanced language model utilizing deep learning techniques to replicate human-like responses to natural language inputs, while Gemini, an advanced AI model developed by Google, showcases multimodal capabilities, understanding text, images, videos, and audio, and excelling in complex tasks across various domains, marking both as significant advancements in the AI landscape [4,5].

ChatGPT and Google Gemini play pivotal roles in patient counseling for Alzheimer’s, Parkinsonism, and MIG. ChatGPT’s advanced language processing abilities enable personalized information delivery, addressing patient queries, and aiding in understanding symptoms and treatments. Meanwhile, Google Gemini’s multimodal capabilities allow enhanced patient education through diverse mediums like images, videos, and audio, fostering greater engagement and comprehension. Together, these AI models assist healthcare professionals in effectively communicating complex concepts and treatment options, empowering patients in managing these neurological conditions.

Traditional patient education materials - such as pamphlets, brochures, and static web pages - often fall short in effectively engaging patients due to their generic content, limited adaptability to individual learning needs, and use of complex medical jargon that can hinder comprehension, especially among those with low health literacy. These resources typically lack interactivity and real-time responsiveness, making it difficult for patients to clarify doubts or receive tailored explanations. In contrast, AI-powered tools like ChatGPT and Google Gemini offer dynamic, personalized, and interactive learning experiences. By leveraging natural language processing and multimodal capabilities, these tools can translate complex medical information into more understandable formats, provide instant responses to patient questions, and adapt explanations based on user input. This positions AI as a promising solution to bridge long-standing gaps in patient education, improving accessibility, comprehension, and ultimately empowering individuals to participate more actively in their healthcare.

There remains a critical need to systematically investigate the effectiveness, usability, and impact of these AI tools within healthcare contexts. This study would be well-rounded by incorporating ChatGPT and Google Gemini conversations in response to patient queries about Alzheimer’s, Parkinsonism, and MIG to gain insights into how AI-driven conversations can enhance patient understanding and support for these conditions.

Aims and objectives

This study aims to compare and analyze the readability, similarity, and reliability of patient education materials generated by ChatGPT and Google Gemini for neurological diseases while providing recommendations for improving AI-generated healthcare information.

## Materials and methods

This study employs a cross-sectional design to evaluate the characteristics of patient education materials generated by large language models (LLMs) for common neurological diseases. The time frame for data collection spanned from February 14, 2024 to February 22, 2024. Given the absence of human participants, the study was deemed exempt from ethical committee approval.

Data collection

Three prevalent neurological diseases, Parkinson’s disease (PD) and AD, along with MIG, were chosen for analysis. Two prominent LLMs, ChatGPT (version 3.5, accessed February 14, 2024) and Google Gemini (version 1.5.2, accessed February 14, 2024), were selected to generate patient education brochures.

Each LLM received a standardized prompt for each disease. The prompt for PD, for example, was “Write a patient education guide for Parkinson’s disease.” Similar prompts were used for AD and MIG. The generated responses from both LLMs for each disease were then collected in a Microsoft Word document and facilitated subsequent analysis.

Data evaluation

The captured responses were meticulously evaluated using various tools, including the Flesch-Kincaid Grade Level and Flesch Reading Ease Score, to provide a quantitative measure of the guides’ readability based on word and sentence count analysis [6].

Originality analysis was done by utilizing the QuillBot Plagiarism Tool (QuillBot, a Learneo, Inc. business, Chicago, Illinois, USA). This included assessing the similarity of the generated content with existing online sources. This analysis provided insights into the originality and potential overlap with pre-existing patient education materials [7].

Lastly, reliability was assessed using the Modified DISCERN score, which was employed to evaluate the trustworthiness of the scientific information presented in the guides. This tool, equipped with specific criteria for assessing healthcare information reliability, offered a structured approach to analyzing the quality of the AI-generated content [8].

Statistical analysis

The data from the graded responses were exported to a Microsoft Excel spreadsheet (Microsoft Corporation, Redmond, WA, USA) to facilitate systematic organization and data cleaning for statistical analysis. R version 4.3.2 was employed for data analysis. An unpaired t-test, suitable for comparing means between independent groups, was used to compare the responses generated by ChatGPT and Google Gemini. A pre-established significance level of p < 0.05 was used to identify statistically significant differences between the AI-generated responses. Additionally, the Pearson correlation coefficient was used to examine the potential correlation between ease score and reliability score, providing insights into the relationship between readability and trustworthiness in the AI-generated materials.

## Results

ChatGPT and Google Gemini LLMs were utilized to create patient education brochures on MIG, PD, and AD, with the aim of assessing the characteristics of AI-generated text for such educational materials.

The mean number of words and sentences did not exhibit statistically significant differences between the two models (Table 1). While both AI models produced similar average words per sentence (p = 0.97), Google Gemini generated significantly shorter texts with fewer sentences (p = 0.04) compared to ChatGPT. Interestingly, there was no significant difference in the average syllables per word (p = 0.28) between the two models. While both AI models scored similarly on the Grade Level metric (p = 0.30), Google Gemini achieved a significantly lower ease score (p = 0.29). This suggests that text generated by Google Gemini might be slightly more complex or require more effort to read compared to ChatGPT outputs. Google Gemini outputs displayed significantly lower similarity scores compared to ChatGPT (p = 0.04). This suggests that text generated by Google Gemini might be more original and less prone to repetitive phrasing compared to outputs from ChatGPT.

**Table 1:** Characteristics of responses generated by ChatGPT and Google Gemini ^*^ p-values <0.05 are considered statistically significant.

Variables	ChatGPT		Google Gemini		p-value (unpaired t-test)
	Mean	SD	Mean	SD	
Words	578.3	96.41	428.7	41.63	0.0989
Sentences	66.0	8.18	48.33	7.23	0.0496^*^
Average words per sentence	8.80	1.3	8.83	0.68	0.9711
Average syllables per word	2.00	0.1	2.13	0.15	0.2846
Grade level	11.43	0.65	13.03	2.02	0.3033
Ease score	28.7	7.1	17.4	13.52	0.2895
Similarity %	22.83	7.60	6.17	5.51	0.0419^*^
Reliability score	3.67	0.58	3.67	0.58	1.000

Pearson correlation coefficients were calculated to assess the relationship between the ease score and reliability score of ChatGPT and Google Gemini. Results showed a moderate negative correlation, albeit statistically insignificant, between ChatGPT and Google Gemini for both ease score (r = -0.2995, P = 0.8064) and reliability score (r = -0.5, P = 0.6667). This suggests a tendency for the ease and reliability scores of Google Gemini to decrease as the corresponding scores of ChatGPT increase, and vice versa, as depicted in Table 2.

**Table 2:** Correlation between ChatGPT and Google Gemini for ease score and reliability score

Variables	Pearson correlation coefficient (r)	p-value
Ease score	-0.2995	0.8064
Reliability score	-0.5	0.6667

## Discussion

A cross-sectional study was conducted to compare responses generated by the two popular AI tools - ChatGPT and Google Gemini - for the common neurological diseases, namely PD, AD, and MIG. Our analysis revealed that AI chatbots generated content for patient education guides created by Google Gemini had fewer sentences when compared with the content created by ChatGPT. Another significant difference in the content by the two AI tools was the similarity percentage, which was low for Google Gemini, indicating more originality in the content.

With the recent AI advancements, it is now becoming an integral part of our lives, revolutionizing every sector, particularly education and healthcare. This is mainly due to their easy-to-use, interactive interface while being free to use. By providing updated, easy-to-comprehend information about complex medical diagnoses, AI chatbots can contribute to transparency and build trust between patients and healthcare providers [9]. Patients can get standard information about their diseases from AI chatbots, which gives physicians more time to engage with individual aspects of the disease with patients [10]. AI chatbots can also be utilized to provide personalized plans and 24/7 support to patients for various lifestyle modifications like physical activity, weight loss, smoking cessation, and treatment or medication adherence [11,12]. In view of the above merits of AI chatbots, we conducted our study in which we compared the AI-generated responses for a patient education guide on the three common neurological diseases - PD, AD, and MIG - using ease, similarity, and reliability scores.

Medical information is often regarded as difficult to read and comprehend, and according to the American Medical Association, patient education material should be at or below the sixth-grade level and use one- or two-syllable words to promote better understanding [13]. In our study, we calculated the Flesch-Kincaid reading ease score for patient education guides for common neurological diseases generated by ChatGPT and Google Gemini and found low ease scores for both AI chatbots, corresponding to a college graduate grade level. Similar studies done to assess the readability of AI-generated content for various other diseases also found them to be difficult to read [14-18]. The poor readability of AI-generated patient information guides highlights an area of improvement in future AI versions.

The similarity score calculated using the QuillBot Plagiarism Tool for patient education guides for common neurological diseases generated using ChatGPT was found to be significantly higher than that of Google Gemini. This finding aligns with other studies that have found high similarity in the AI chatbot-generated medical educational content [18,19]. The content generated by AI chatbots is based on large datasets of existing text that were used in training the program. This can lead to various issues such as high similarity to the existing texts, inaccuracies, and biases, depending on the quality of the original training content [18-20]. Although for scientific literature, the maximum similarity score cutoff is around 10-15%, in our study, the mean similarity scores of patient education guides generated by ChatGPT were found to be 22.83 and 6.17 for Google Gemini. Mondal et al. found a maximum similarity score of 37% for AI chatbot-generated patient educational material [19].

This difference in training corpora could influence not only response length but also the degree of originality and could explain the observed difference in response length and originality between Google Gemini and ChatGPT [19].

To assess the reliability of the AI chatbot-generated patient educational guides for common neurological diseases, a modified DISCERN score was used [21]. It has been widely used in the literature to assess the reliability of publicly available online healthcare information [15]. It consists of five questions, and each patient education guide was scored from 1 to 5 based on those questions. The mean reliability score for patient education guides generated by ChatGPT and Google Gemini was 3.67. A recent study comparing DISCERN scores of ChatGPT-generated health information with those published on websites reported them to be similar [4].

Limitations

This study has several limitations. First, we utilized only two AI chatbots - ChatGPT (version 3.5) and Google Gemini (version 1.5.2) - to generate patient educational guides, despite the availability of other public-facing AI tools that may perform differently. Second, we employed ChatGPT version 3.5, which, while freely accessible to the public, is not the most advanced iteration currently available. It is also important to note that these AI models require regular updates to reflect the most recent medical advancements. Third, we did not compare the AI-generated outputs against standardized patient education brochures, such as those published by reputable organizations like the Parkinson’s Foundation. Including such materials could have provided a meaningful benchmark for evaluating content quality and reliability. Lastly, although our study emphasized understandability and length, it did not evaluate the medical accuracy or contextual meaning of the generated content in depth. Moreover, while originality is often valued in content generation, health education relies on evidence-based, standardized information, and generating highly novel content may not always be appropriate or necessary in this context.

## Conclusions

The present study compared patient educational guides for common neurological conditions generated by ChatGPT and Google Gemini, revealing that both tools produced content that was generally difficult for the public to read and understand, despite having comparable levels of reliability. These findings underscore the need for future iterations of AI chatbots to prioritize clarity, simplicity, and up-to-date healthcare information, along with ongoing oversight to ensure content accuracy and appropriateness.

This study highlights the importance of critically evaluating AI-generated content before recommending it to patients. As AI tools become more integrated into healthcare communication, clinicians can play a pivotal role in guiding patients toward trustworthy resources and supplementing chatbot-generated material with professional insight. Ultimately, understanding the strengths and limitations of these tools can help providers enhance patient education, support shared decision-making, and improve health literacy across diverse populations.

## Appendices

**Table 3:** Patient education guide generated for "Migraine"

Write a patient education guide for “Migraine”.	
ChatGPT	Google Gemini
Title: Understanding and Managing Migraines: A Patient Education Guide Introduction: Living with migraines can be challenging, but with the right knowledge and management strategies, you can gain better control over your symptoms and improve your quality of life. This patient education guide aims to provide you with essential information about migraines, their triggers, symptoms, and effective management techniques. Section 1: What is a Migraine? A migraine is a neurological disorder characterized by recurrent, intense headaches that often come with other symptoms such as nausea, sensitivity to light and sound, and visual disturbances. Migraines can significantly impact your daily life, but understanding the condition is the first step towards managing it effectively. Section 2: Recognizing Migraine Triggers Identifying and avoiding triggers can help prevent migraines. Common triggers include: 1. **Stress:** Practice stress management techniques such as deep breathing, meditation, and yoga. 2. **Dietary Factors:** Identify and avoid specific food triggers, such as chocolate, caffeine, and certain additives. 3. **Sleep Patterns:** Maintain a consistent sleep schedule, ensuring you get enough quality sleep. 4. **Hormonal Changes:** Women may experience migraines related to hormonal fluctuations; tracking menstrual cycles can be helpful. Section 3: Migraine Symptoms Recognizing the symptoms of a migraine is crucial for early intervention. Common symptoms include: 1. **Headache:** Typically throbbing and on one side of the head. 2. **Aura:** Visual disturbances or other sensory changes preceding the headache. 3. **Nausea and Vomiting:** Gastrointestinal symptoms may accompany the headache. 4. **Sensitivity to Light and Sound:** Migraine sufferers often find light and sound intolerable during an attack. Section 4: Seeking Professional Help If you suspect you have migraines, it's essential to consult a healthcare professional. A thorough medical evaluation can confirm the diagnosis and help create an effective treatment plan. Your healthcare provider may recommend medications, lifestyle modifications, or a combination of both. Section 5: Lifestyle Modifications and Management Strategies 1. **Regular Exercise:** Engage in regular, moderate exercise to help reduce the frequency and intensity of migraines. 2. **Hydration:** Stay well-hydrated, as dehydration can trigger migraines. 3. **Regular Sleep Patterns:** Ensure you get enough sleep and maintain a consistent sleep schedule. 4. **Healthy Diet:** Adopt a balanced diet, avoiding known trigger foods. 5. **Stress Management:** Practice relaxation techniques, such as deep breathing, meditation, or progressive muscle relaxation. Section 6: Medications for Migraine Management Your healthcare provider may prescribe medications to manage migraines, including: 1. **Acute Medications:** Taken during an attack to relieve symptoms. 2. **Preventive Medications:** Taken regularly to reduce the frequency and severity of migraines. Section 7: Keeping a Migraine Diary Maintain a migraine diary to track potential triggers, symptoms, and the effectiveness of treatments. This can provide valuable insights and help you and your healthcare provider develop a more personalized management plan. Conclusion: Managing migraines requires a holistic approach that combines lifestyle modifications, stress management, and, when necessary, medications. With proper education and support, you can take control of your migraines and improve your overall well-being. Remember to consult with your healthcare provider for personalized advice tailored to your specific needs.	Migraine: Understanding and Managing Your Headaches Migraine is a common neurological disorder affecting millions of people worldwide. It manifests as intense, throbbing headaches often accompanied by various other symptoms like nausea, vomiting, light and sound sensitivity, and dizziness. While debilitating, understanding its causes and management options can empower you to better manage your condition and improve your quality of life. Understanding Migraine: What is it? Migraine is not just a "bad headache." It's a complex neurological disorder involving changes in brain signals, blood flow, and chemical pathways. Types of Migraine: There are different types, including migraine with aura (visual disturbances before headache), migraine without aura (headache without visual symptoms), and chronic migraine (headaches occurring on 15 or more days per month). Triggers: Identifying your triggers like stress, lack of sleep, certain foods, hormonal changes, or environmental factors is crucial for prevention. Managing Migraine: Treatment options: Acute medications: Over-the-counter pain relievers and prescription medications can help manage attack symptoms. Preventive medications: Daily medications can reduce the frequency and severity of attacks. Lifestyle changes: Regular sleep, stress management, relaxation techniques, and a healthy diet can significantly impact migraine. Complementary therapies: Acupuncture, biofeedback, and massage therapy may offer some relief for some individuals. Living with Migraine: Managing stress: Techniques like yoga, meditation, and deep breathing can help reduce stress, a common trigger. Maintaining a healthy lifestyle: Regular sleep, exercise, and a balanced diet contribute to overall well-being and may impact migraines. Developing a headache diary: Tracking your headaches, symptoms, and potential triggers helps identify patterns and inform treatment decisions. Finding support: Connecting with other individuals with migraine through support groups or online communities can offer valuable advice and understanding. Remember: Consult your doctor: Discuss your specific needs and develop a personalized management plan. Be patient: Finding the right treatment combination may take time and experimentation. Don't suffer in silence: There are effective treatments available, and you deserve to feel better. Additional Resources: American Migraine Foundation: https://americanmigrainefoundation.org/ American Headache Society: https://americanheadachesociety.org/ National Headache Foundation: https://headaches.org/ Migraine Research Foundation: https://americanmigrainefoundation.org/ Disclaimer: This guide is for informational purposes only and does not constitute medical advice. Please consult your healthcare professional for diagnosis and treatment of your individual condition.

**Table 4:** Patient education guide generated by the chatbots for "Parkinson's disease"

Write a patient education guide for “Parkinson disease”		
AI	ChatGPT	Google Gemini
Response	Title: Understanding Parkinson's Disease: A Patient Education Guide Introduction: Parkinson's disease is a neurological disorder that affects movement. It develops gradually, often starting with subtle symptoms that may be overlooked. Understanding the condition, its symptoms, and available treatments is crucial for managing Parkinson's disease effectively. This guide aims to provide you with essential information about Parkinson's disease, its symptoms, diagnosis, treatment options, and strategies for living well with the condition. What is Parkinson's Disease? Parkinson's disease is a progressive disorder of the nervous system that primarily affects movement. It occurs when nerve cells (neurons) in the brain gradually break down or die. These neurons produce dopamine, a chemical messenger responsible for transmitting signals that control movement and coordination. As dopamine levels decrease, movement becomes impaired, leading to the characteristic symptoms of Parkinson's disease. Symptoms: The symptoms of Parkinson's disease can vary from person to person and may change over time. Common symptoms include: 1. Tremors: Involuntary shaking, usually beginning in a limb, often while at rest. 2. Bradykinesia: Slowed movement, making simple tasks difficult and time-consuming. 3. Muscle rigidity: Stiffness or tightness in the muscles, reducing flexibility and causing discomfort. 4. Postural instability: Impaired balance and coordination, increasing the risk of falls. 5. Changes in speech: Softening of the voice, slurred speech, or hesitation before speaking. 6. Bradyphrenia: Slowed thinking and cognitive impairment, affecting memory and decision-making. 7. Non-motor symptoms: These can include depression, anxiety, sleep disturbances, constipation, and loss of sense of smell. Diagnosis: Diagnosing Parkinson's disease can be challenging, as there is no specific test for it. A diagnosis is typically based on medical history, a thorough physical examination, and the presence of characteristic symptoms. In some cases, brain imaging studies such as MRI or CT scans may be used to rule out other conditions. A neurologist, a doctor specializing in disorders of the nervous system, usually makes the diagnosis. Treatment: While there is no cure for Parkinson's disease, several treatment options are available to manage symptoms and improve quality of life: 1. Medications: Dopamine-replacement drugs, such as levodopa, can help alleviate motor symptoms by replenishing dopamine levels in the brain. 2. Physical therapy: Exercises and physical therapy techniques can improve mobility, flexibility, and balance. 3. Occupational therapy: Occupational therapists can suggest strategies and assistive devices to help with daily activities. 4. Speech therapy: Speech therapists can work with individuals to improve communication skills and address speech difficulties. 5. Deep brain stimulation (DBS): In some cases, surgically implanted electrodes can help regulate abnormal brain activity and reduce symptoms. 6. Lifestyle modifications: Regular exercise, a healthy diet, adequate sleep, and stress management techniques can all contribute to better symptom management and overall well-being. Living with Parkinson's Disease: Living with Parkinson's disease presents unique challenges, but with proper management and support, many individuals can lead fulfilling lives. Here are some tips for living well with Parkinson's: 1. Educate yourself: Learn as much as you can about Parkinson's disease to better understand your condition and treatment options. 2. Stay active: Regular exercise, such as walking, swimming, or tai chi, can help improve mobility, balance, and mood. 3. Eat a balanced diet: Aim for a diet rich in fruits, vegetables, whole grains, and lean proteins to support overall health and well-being. 4. Seek support: Joining a support group or connecting with others living with Parkinson's disease can provide valuable emotional support and practical advice. 5. Manage medications: Take medications as prescribed and keep track of any side effects or changes in symptoms. 6. Stay engaged: Pursue hobbies, interests, and social activities to stay mentally and emotionally stimulated. 7. Communicate with your healthcare team: Keep your healthcare providers informed about any changes in symptoms or concerns you may have. Conclusion: Parkinson's disease is a complex condition that requires ongoing management and support. By understanding the symptoms, diagnosis, treatment options, and strategies for living well, individuals with Parkinson's disease can take an active role in their care and maintain the best possible quality of life. Working closely with healthcare providers, staying informed, and seeking support from loved ones and peers can make a significant difference in managing Parkinson's disease effectively.	Understanding Parkinson's Disease: A Patient Education Guide Parkinson's disease can be a complex and overwhelming diagnosis, but understanding the condition is key to managing your symptoms and living a fulfilling life. This guide provides an overview of Parkinson's, its symptoms, treatment options, and resources to help you navigate your journey. What is Parkinson's Disease? Parkinson's is a progressive neurological disorder that affects the brain, specifically an area responsible for movement control. It causes a loss of nerve cells that produce dopamine, a vital chemical for smooth movement. Symptoms: Tremor: A rhythmic shaking, often starting in one hand and progressing. Rigidity: Muscle stiffness and reduced flexibility. Bradykinesia: Slowness of movement, making daily activities challenging. Postural instability: Difficulty maintaining balance and increased risk of falls. Non-motor symptoms: Sleep disturbances, fatigue, depression, cognitive changes, and more. Diagnosis: There is no single test for Parkinson's. Diagnosis involves a doctor's evaluation, including your medical history, neurological examination, and sometimes imaging tests. Treatment: While there is no cure, various treatments can help manage symptoms and improve quality of life. These include: Medications: Levodopa is the most common medication, replenishing dopamine levels. Other drugs address specific symptoms. Deep brain stimulation (DBS): Electrical stimulation of the brain for advanced cases. Physical therapy: Helps maintain mobility, flexibility, and balance. Occupational therapy: Trains for daily activities and adapts your environment for independence. Speech therapy: Improves communication if speech is affected. Lifestyle Changes: Exercise: Regular physical activity can improve motor function, balance, and overall well-being. Healthy diet: A balanced diet with fruits, vegetables, and whole grains may offer benefits. Stress management: Techniques like meditation and yoga can help manage stress and improve sleep. Support groups: Connecting with others with Parkinson's can provide valuable emotional and practical support. Living with Parkinson's: Talk to your doctor regularly: Discuss your symptoms, treatment options, and any concerns. Stay informed: Learn about Parkinson's and its management through reliable sources. Join a support group: Connect with others who understand your journey. Advocate for yourself: Ask questions, express your needs, and participate in your treatment decisions. Focus on quality of life: Engage in activities you enjoy, stay connected with loved ones, and maintain a positive outlook. Additional Resources: The Michael J. Fox Foundation: [https://www.michaeljfox.org/] The American Parkinson Disease Association (APDA): [https://www.apdaparkinson.org/] The National Institute of Neurological Disorders and Stroke (NINDS): [[invalid URL removed]] Remember: This guide is a starting point. Consult your doctor for personalized advice and support throughout your journey with Parkinson's. Disclaimer: This information is not intended as a substitute for professional medical advice. Always consult with your doctor for diagnosis and treatment planning.

**Table 5:** Patient education guides generated for "Alzheimer disease"

Write a patient education guide for “Alzheimer disease”	
ChatGPT	Google Gemini
Title: Understanding Alzheimer's Disease: A Patient Education Guide Introduction: Dear Patient, This guide is designed to provide you with valuable information about Alzheimer's disease, a condition that affects memory, thinking, and behavior. Knowledge is a powerful tool, and by understanding Alzheimer's, you and your loved ones can navigate this journey with greater awareness and compassion. Table of Contents: 1. **Overview of Alzheimer's Disease:** - Definition and Basics - Causes and Risk Factors 2. **Symptoms:** - Early Signs - Progression of Symptoms 3. **Diagnosis:** - Medical History and Physical Examination - Cognitive and Memory Tests - Imaging and Laboratory Tests 4. **Treatment Options:** - Medications - Non-Pharmacological Interventions - Lifestyle Modifications 5. **Living with Alzheimer's:** - Communication Strategies - Daily Routine and Activities - Safety Precautions at Home 6. **Support for Caregivers:** - The Role of Caregivers - Seeking Professional Support - Self-Care for Caregivers 7. **Planning for the Future:** - Legal and Financial Planning - Advance Directives - Long-Term Care Options 8. **Community Resources:** - Alzheimer's Associations and Support Groups - Educational Programs - Online Resources Section 1: Overview of Alzheimer's Disease Alzheimer's disease is a progressive brain disorder that impacts memory, cognitive function, and behavior. It is the most common cause of dementia, a term used to describe a decline in mental abilities that interfere with daily life. The exact cause of Alzheimer's is not fully understood, but a combination of genetic, environmental, and lifestyle factors may contribute to its development. Section 2: Symptoms Early signs of Alzheimer's include forgetfulness, difficulty solving problems, and confusion about time and place. As the disease progresses, individuals may experience challenges in communication, mood swings, and changes in personality. Recognizing these symptoms early allows for better management and planning. Section 3: Diagnosis Diagnosing Alzheimer's involves a thorough examination, including medical history, cognitive tests, and sometimes imaging studies. Early diagnosis helps in developing a suitable care plan and may involve ruling out other conditions that can mimic Alzheimer's symptoms. Section 4: Treatment Options While there is no cure for Alzheimer's, certain medications can help manage symptoms and slow down their progression. Non-pharmacological interventions such as cognitive stimulation, physical exercise, and a healthy diet also play a crucial role in maintaining overall well-being. Section 5: Living with Alzheimer's Adapting to life with Alzheimer's involves finding effective communication strategies, establishing a daily routine, and ensuring a safe home environment. Engaging in activities that stimulate the mind and staying socially connected can enhance quality of life. Section 6: Support for Caregivers Caregivers play a vital role in supporting individuals with Alzheimer's. It is essential for caregivers to seek professional support, join support groups, and prioritize their own well-being to provide the best care possible. Section 7: Planning for the Future Planning for the future includes legal and financial considerations, discussing preferences for care, and exploring long-term care options. Advance directives and open communication within families are crucial aspects of future planning. Section 8: Community Resources Various resources, including Alzheimer's associations, support groups, and educational programs, can provide valuable information and assistance. Online platforms also offer a wealth of knowledge and connection with others facing similar challenges. Conclusion: Understanding Alzheimer's disease empowers individuals and their families to navigate the journey with knowledge and compassion. By accessing appropriate resources, seeking support, and planning for the future, individuals with Alzheimer's and their caregivers can enhance the quality of life and maintain a sense of dignity throughout the progression of the disease.	Understanding Your Journey: A Guide to Alzheimer's Disease What is Alzheimer's Disease? Alzheimer's disease is the most common form of dementia, affecting millions worldwide. It's a progressive brain disorder that gradually damages memory, thinking, and eventually, behavior. While there's no cure, understanding this disease empowers you to make informed decisions and navigate its challenges. Early Signs and Recognizing Changes: Alzheimer's symptoms often begin subtly, like forgetting names, misplaced belongings, or mild confusion. As the disease progresses, changes become more noticeable: Memory: Difficulty remembering recent events, conversations, or familiar places. Thinking and Reasoning: Struggles with problem-solving, decision-making, or following complex instructions. Language: Difficulty finding the right words, struggling with conversations, or repetitive speech. Behavior: Personality changes, mood swings, anxiety, or challenges with daily tasks. Seeking Diagnosis and Support: If you're experiencing these changes, it's important to consult your doctor. They can conduct cognitive assessments, medical history review, and imaging tests to reach a diagnosis. Early diagnosis allows for timely intervention and planning. Remember, there's no shame in seeking help, and it can empower you to manage the disease effectively. Living with Alzheimer's: Living with Alzheimer's requires adaptation and support. Here are some tips: Establish Routines: Consistent routines provide stability and predictability, making daily tasks easier to manage. Memory Aids: Utilize calendars, planners, labeled organizers, and technological tools to support memory. Stay Engaged: Participate in activities you enjoy, like social gatherings, hobbies, or physical activities. Connect with Others: Join support groups, connect with friends and family, and seek professional help when needed. Treatment and Management: While there's no cure, various treatment options can help manage symptoms and improve quality of life. These include: Medications: Medications can slow the disease's progression and manage symptoms like memory loss and confusion. Therapies: Cognitive stimulation therapy, music therapy, and occupational therapy can help maintain cognitive function and daily living skills. Lifestyle Changes: A healthy diet, regular exercise, and good sleep hygiene can positively impact brain health. Remember, You're Not Alone: Living with Alzheimer's can be challenging, but there's a wealth of support available. Utilize resources from organizations like the Alzheimer's Association, National Institute on Aging, and local dementia support groups. Remember, you're not alone in this journey. Additional Resources: Alzheimer's Association: https://www.alz.org/ National Institute on Aging: https://www.nia.nih.gov/health/alzheimers-and-dementia Alzheimer's Society: https://www.alzheimers.org.uk/ Dementia Support: https://alzheimer.ca/en Disclaimer: This guide is for informational purposes only and should not be considered medical advice. Please consult with your healthcare provider for diagnosis and treatment specific to your situation. Remember, knowledge is power. By educating yourself, seeking support, and embracing a proactive approach, you can navigate this journey with strength and resilience.
